# A Critical Narrative Review Appraisal of the 2025–2030 Dietary Guidelines: Scientific Strengths, Conceptual Gaps, and Overlooked Dimensions of Sustainability and Health Equity

**DOI:** 10.3390/nu18071040

**Published:** 2026-03-25

**Authors:** Dimitrios Papandreou, Azza Alsuwaidi, Zainab Taha, Constantinos Giaginis, Georgios K. Vasios, Eleni P. Andreou

**Affiliations:** 1 Department of Clinical Nutrition and Dietetics, College of Health Sciences, University of Sharjah, Sharjah 27272, United Arab Emirates; u24102805@sharjah.ac.ae; 2 Department of Health Sciences, CNHS, Zayed University, Abu Dhabi 144534, United Arab Emirates; zainab.taha@zu.ac.ae; 3 Department of Food Science and Nutrition, School of Environment, University of the Aegean, Mitropoliti Ioakeim 2, GR81400 Myrina, Limnos, Greece; vasios@aegean.gr; 4 Department of Life Sciences, School Life and Health Sciences, University of Nicosia, 1700 Nicosia, Cyprus; andreou.el@unic.ac.cy

**Keywords:** guidelines 2025–2030, ultra-processed foods, sustainable diet, health equity, nutrition policy

## Abstract

The 2025–2030 Dietary Guidelines introduce an important shift in public health nutrition, emphasizing minimally processed foods, higher protein intake, greater inclusion of full-fat dairy, and a food-based advice centered on “real food” consumption. While several of these recommendations align with accumulating evidence, particularly the discouragement of ultra-processed foods and added sugars, substantial concerns remain regarding their internal coherence, population-level applicability, risk of misinterpretation, as well as environmental footprint. This critical narrative review evaluates whether the scope, emphasis, and framing of the new guideline components are proportionate to the strength, consistency, and context of the underlying evidence. Using a novel framework that distinguishes between nutritional adequacy, optimization, and therapeutic application, we assess the scientific coherence of key recommendations. A structured literature search was conducted in PubMed, Scopus, and Web of Science focusing on systematic reviews and meta-analyses of randomized controlled trials and large prospective cohort studies relevant to the updated guidelines. Particular attention is given to protein and saturated fat intakes, carbohydrate restriction in chronic disease, and the balance between simplification and scientific precision. Overall, the new guidelines represent a positive shift toward food-based recommendations; however, clearer differentiation between population-level guidance and context-specific interventions is required to preserve scientific rigor, reduce misinterpretation, and enhance public health relevance.

## 1. Introduction

Dietary guidelines are a cornerstone of public health nutrition, influencing national policy, institutional food provision, clinical advice, and broader food system priorities. At the same time, they must navigate inherent scientific uncertainty, wide variation across populations, and the practical challenges of large-scale implementation [1,2]. In contrast to clinical practice guidelines which target well-defined patient groups and often draw on narrowly controlled evidence frameworks dietary guidelines are designed for diverse populations. Their development must therefore strike a careful balance between scientific rigor and clear, accessible, and equitable communication. This broader mandate underscores the importance of clearly distinguishing between evidence that supports basic nutritional adequacy, evidence that informs optimal intake for specific subgroups, and evidence that is relevant primarily within therapeutic or clinical settings [2,3].

Over recent decades, dietary guidelines have faced growing criticism for their heavy reliance on nutrient-focused, reductionist frameworks. These approaches often consider individual nutrients in isolation from the foods and overall dietary patterns in which they are consumed [1]. While this method has been instrumental in identifying nutrient deficiencies and key cardiometabolic risk factors, it has also led to public confusion unintended dietary substitutions, and policy messages that are difficult to translate into real-world eating habits [4,5]. In response, recent guideline processes have begun to shift their emphasis toward dietary patterns and the food matrix, as well as considering environmental, socioeconomic, and implementation realities.

**Figure 1:**
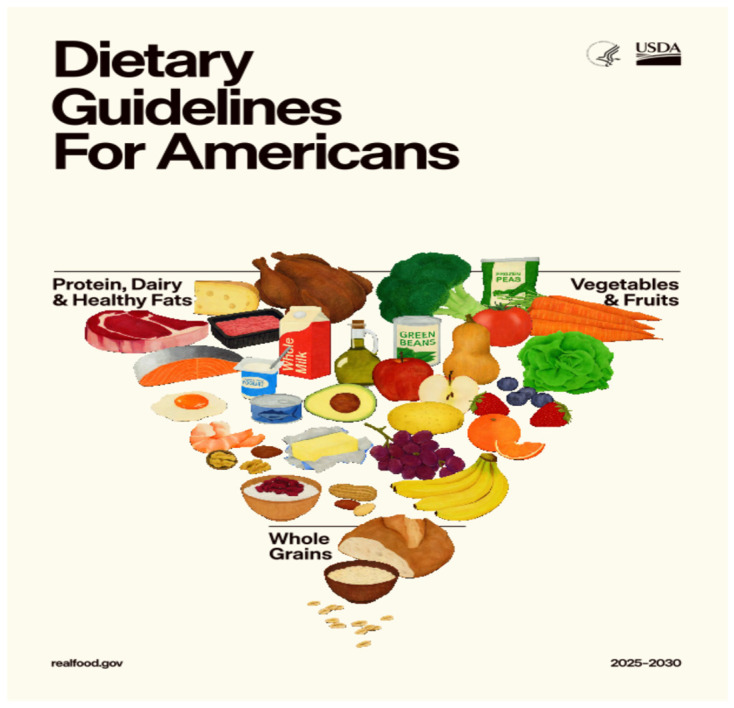
USDA 2026. Conceptual illustration of the 2025–2030 Dietary Guidelines.

The 2025–2030 Dietary Guidelines (Figure 1) mark the culmination of this evolving approach, with a clear emphasis on the consumption of “real foods” and a stronger stance against highly processed products. There is now a significant growing body of evidence linking ultra-processed food intake to increased risks of cardiometabolic disease, cancer, and all-cause mortality across diverse populations [6,7]. Ultra-processed foods often provide cost-effective nutrients for disadvantaged populations and environmental sustainability, where wholesale reduction could increase resource demands [8]. This reframing is further supported by a new emphasis on gut health, which appropriately recognizes the role of dietary patterns in maintaining a balanced gut microbiome. This represents a positive development, reflecting a more holistic understanding of the interconnected physiological processes that influence overall health. At the same time, the guidelines depart in several important ways from previous editions, including the population-level encouragement of higher protein intake, a more permissive position on full-fat dairy and certain animal fats, and explicit recognition of lower-carbohydrate dietary patterns in the management of chronic disease. While the Mediterranean diet is rich in unsaturated fats from olive oil and nuts, the US guidelines’ permissive framing of butter and tallow as comparable “healthy fats” is inconsistent with this evidence base. Similarly, the Mediterranean pattern emphasizes plant-based protein sources, fish, and poultry, with red meat consumed infrequently and in small amounts that increases longevity and well-being [9]. This contrasts sharply with the US guidelines’ prominent placement of red meat as a primary protein source without upper limits or risk qualification. Finally, the Mediterranean diet’s foundation is high-fiber, minimally processed whole grains and legumes, an emphasis on quality that is largely absent from the US guidelines’ discussion of carbohydrates [10]. This comparison underscores how the 2025–2030 guidelines deviate from globally recognized, evidence-based patterns of eating. Collectively, all these changes reflect an effort to incorporate emerging scientific evidence and to address longstanding critiques of the rigidity of earlier guideline frameworks. However, translating ongoing scientific debate into guidance suitable for entire populations remains inherently challenging. Nutrition research is marked by diverse study designs, generally modest effect sizes, long time horizons for the development of chronic disease, and an unavoidable dependence on observational evidence for many key dietary exposures [3,11].

As a result, the level of evidence required to support population-level recommendations must be set with care. Guidance grounded in moderate, conditional, or context-specific evidence such as findings from studies of older adults, individuals with chronic disease, or weight-loss interventions may not be suitable for universal application. Such as recommendations require clear framing and appropriately qualification to prevent misinterpretation [2,12].

Moreover, dietary guidelines function not only as scientific syntheses but also as normative policy tools. The way recommendations are framed shapes food procurement policies, nutrition assistance programs, clinical practice norms, and public perceptions of dietary risk. While simplified messaging may improve accessibility and reach, it can also encourage selective or distorted interpretations particularly in media and commercial settings where important nuances are often omitted [1]. This concern is especially pronounced for guidance on protein intake and dietary fats, areas in which the scientific evidence remains mixed and public debate is highly polarized.

Within this context, critical appraisal of dietary guidelines is essential. Beyond asking whether individual recommendations are supported by some level of evidence, it is necessary to examine whether the strength, consistency, and real-world relevance of that evidence justify their presentation as population-wide guidance. It is also important to assess the internal coherence of recommendations, their alignment with established biological and causal pathways, and the potential for unintended dietary consequences arising from overly simplified or ambiguous messaging. However, translating emerging nutrition evidence into population-wide dietary guidelines requires clear differentiation between evidence derived from specific clinical or subgroup contexts and evidence that is sufficiently robust to support universal public health recommendations.

The objective of this review is to critically examine the scientific coherence and evidentiary basis of the 2025–2030 Dietary Guidelines. Moving beyond a descriptive summary, we introduce and apply an analytical framework that distinguishes between evidence for (a) nutritional adequacy (preventing deficiency), (b) optimization (enhancing function in specific groups), and (c) therapeutic application (managing disease). By evaluating the guidelines through this lens, we aim to provide policymakers, researchers, and practitioners with a clearer understanding of where the evidence is being appropriately applied and where it is being overextended, ultimately contributing to a more rigorous and complex process for future dietary guidance. To achieve this, our analysis is structured around three interconnected criteria. First, we examine the “scope” of the recommendation, namely whether it is intended for the general, healthy population. Second, we analyze its “framing”, focusing on how the recommendation is presented and whether it accurately reflects the strength and complexity of the underlying evidence. Finally, we consider its “population-level applicability”, assessing whether the recommendation is actionable and appropriate for a diverse population, while considering factors such as accessibility, cost, and the potential for misinterpretation.

## 2. Materials and Methods

This critical narrative review was conducted to examine the scientific soundness, strength of evidence, and relevance to the general population of the 2025–2030 Dietary Guidelines. The intention was not to provide an exhaustive summary of the literature, but to assess whether the scope, emphasis, and framing of the recommendations are justified by the quality, consistency, and overall body of available evidence.

A structured literature search was conducted in PubMed, Scopus, and Web of Science. Search terms included both controlled vocabulary and free-text keywords related to dietary guidelines, ultra-processed foods, protein intake, dietary fats and saturated fats, carbohydrate restriction, chronic disease, and public health nutrition. Reference lists from relevant reviews and consensus documents were also examined to identify additional key studies. Priority was given to systematic reviews and meta-analyses of randomized controlled trials where available, alongside large prospective cohort studies for long-term outcomes that are not readily captured in intervention trials. Authoritative consensus reports from major national and international bodies, including the National Academies of Sciences, Engineering, and Medicine, the World Health Organization, and the American Heart Association, were also included. Individual randomized trials were considered when they offered important mechanistic or comparative insights beyond existing syntheses, while mechanistic studies were selectively used to support biological plausibility when interpreting observational findings.

Evidence appraisal in this review was deliberately qualitative and interpretive rather than algorithmic. Studies were assessed based on the consistency of findings across different populations, the magnitude and direction of reported effects, biological plausibility, potential sources of bias related to study design, and their applicability to the general population as opposed to specific subgroups, such as older adults, individuals with chronic disease, or those engaged in intentional weight loss. Emphasis was placed on differentiating evidence that supports basic nutritional adequacy from evidence that underpins optimization or therapeutic use. Importantly, the review explicitly examined whether evidence generated in clinical or context-specific settings had been extended to population-level recommendations within the guidelines. Where such extrapolation was evident, the adequacy of its justification and the presence or absence of appropriate qualifying language were carefully evaluated. The analysis further considered the internal consistency of recommendations across guideline domains, their alignment with established causal frameworks such as lipid-mediated cardiovascular risk, and the potential for unintended dietary consequences resulting from overly simplified or ambiguous messaging. A formal systematic review approach was not adopted, as the primary aim was not to produce a comprehensive synthesis of effect estimates, but to critically assess the coherence, proportionality, and policy interpretation of the evidence base informing the guidelines.

## 3. Critical Evaluation of Major Guideline Components

### 3.1. Minimally Processed Foods and Ultra-Processed Food Restriction

The clear recommendation to limit ultra-processed foods stands out as the most strongly evidence-based element of the 2025–2030 Dietary Guidelines and one of the few areas where the guidance closely reflects the direction, consistency, and overall weight of current scientific evidence. Across multiple large prospective cohort studies and reviews, higher intake of ultra-processed foods is consistently associated with increased risks of obesity, type 2 diabetes, cardiovascular disease, certain cancers, and all-cause mortality, even after extensive adjustments for sociodemographic factors, lifestyle behaviors, and overall diet quality [6,7]. Notably, these relationships have been observed across different populations, dietary assessment methods, and operational definitions of ultra-processed foods, indicating that the findings are unlikely to be explained solely by residual confounding or measurement limitations.

Although much of the evidence in this area is derived from observational cohort studies, the well-recognized limitations of nutritional epidemiology do not diminish the public health relevance of these findings. The presence of dose–response relationships, appropriate temporal sequencing, and consistent results across independent cohorts strengthens causal inference, particularly when viewed alongside experimental data showing increased energy intake and weight gain under ultra-processed dietary conditions [13]. In addition, these associations are supported by a coherent set of biological mechanisms, including higher energy density; disruption of food structure and mastication; rapid glycemic responses; impaired satiety signaling; unfavorable alterations in gut microbiota; and exposure to emulsifiers, sweeteners, and other additives with potential metabolic and inflammatory effects [5]. In this context, the guidelines strong discouragement of ultra-processed foods is not only scientifically justified but also aligned with broader public health goals to shift dietary patterns toward minimally processed, nutrient dense foods such as vegetables, fruits, legumes, whole grains, nuts, and seeds [14].

The guidelines’ focus on reducing ultra-processed food intake represents one of the few areas where simplified public health messaging appears well aligned with the strength of the underlying evidence. Consistent associations have been documented across large prospective cohort studies, umbrella reviews, and controlled experimental investigations [6,7,13]. Given the widespread exposure to ultra-processed foods in many populations and the limited success of earlier nutrient-centered strategies, a framework that prioritizes food processing may offer greater practical relevance and policy impact than more reductionist approaches [5]. The clarity and consistency of evidence in this domain stand in contrast to several other guideline components, where recommendations appear to extend beyond similarly robust evidentiary foundations. This asymmetry underscores the importance of applying uniform evidence hierarchies across topics, particularly when translating conditional or context-specific findings into population-level guidance [3].

This contrast becomes particularly clear in the introduction of per-meal limits for added sugar. While strong evidence supports reducing added or free sugar intake due to well-established links with dental caries, excess energy consumption, weight gain, and cardiometabolic risk, translating this evidence into meal-specific thresholds represents a notable departure from established public health practice [15]. International guidelines have traditionally expressed sugar intake targets as a proportion of total daily energy intake, reflecting substantial variation in meal frequency, cultural eating patterns, and the distribution of energy intake across the day. At present, there is limited evidence to suggest that per-meal sugar thresholds provide greater predictive value for health outcomes than measures based on total daily intake.

The lack of a clearly articulated methodological justification for adopting a per-meal framework raises concerns about arbitrariness and reproducibility. From a practical standpoint, meal-based sugar limits may be challenging to implement in clinical counseling, nutrition education, and institutional food services, where dietary advice is typically organized around daily or weekly intake goals. In addition, such framing may unintentionally divert attention from overall dietary quality and cumulative sugar exposure, potentially encouraging compensatory intake across meals. In the absence of stronger empirical support and clearer rationale, the per-meal approach risks weakening the clarity and real-world applicability of an otherwise well-supported recommendation to reduce added sugar consumption [16,17].

### 3.2. Protein Intake: Adequacy Versus Optimization

In contrast to the well-supported recommendation on ultra-processed foods, the evidence for a population-wide increase in protein intake lacks similar consistency and directness, representing a potential over-extension of conditional evidence. The recommendation to increase protein intake to 1.2–1.6 g/kg/day for the general population marks a substantial departure from long-standing nutritional requirements and therefore merits careful evaluation. Furthermore, presenting protein intake as a target range (1.2–1.6 g/kg) without placing it within the context of total energy intake may be misleading. For individuals already consuming an energy-adequate diet, such levels of protein represent a substantial contribution to total caloric intake. If this increase is not balanced by a corresponding reduction in other macronutrients, it could gradually lead to a positive energy balance and, over time, contribute to weight gain. The current Recommended Dietary Allowance (RDA) of 0.8 g/kg/day was established to meet the needs of nearly all healthy adults and is based on nitrogen balance methodology, supported by decades of metabolic research and population-level evidence [2]. Importantly, the RDA is designed to ensure protein adequacy and prevent deficiency, rather than optimize body composition, physical performance, or other functional outcomes.

Much of the evidence cited in support of higher protein intakes originates from specific physiological or clinical settings. Research in older adults indicates that intakes modestly above the RDA may help mitigate anabolic resistance and slow age-related declines in muscle mass and function, particularly when combined with resistance training [18]. Higher protein consumption has also been shown to aid in the preservation of lean mass and enhance satiety during periods of energy restriction in weight-loss interventions [12]. Similarly, resistance-trained individuals may benefit from elevated protein intakes to support muscle protein synthesis under conditions of high training demand. These recommendations likely reflect growing concern about sarcopenia, obesity, and dietary satisfaction in aging populations, as well as efforts to address declining muscle mass and metabolic health across the lifespan; however, such concerns do not automatically justify the extension of subgroup-specific evidence to universal population targets.

Critically, extending these context-specific findings to population-wide recommendations is not well supported by the current evidence. To date, large-scale randomized controlled trials in generally healthy adults have not yet demonstrated clear long-term advantages of higher protein intakes in terms of morbidity, mortality, or sustained cardiometabolic risk reduction compared with more moderate protein consumption within high-quality dietary pattern [12]. Observational evidence linking higher protein intake to favorable health outcomes is also mixed and appears strongly influenced by protein source, overall dietary pattern, and the extent to which protein displaces other macronutrients, thereby limiting causal inference [2,12]. Consequently, the evidence base for recommending elevated protein intake as a normative target for the general population remains weak, particularly in the absence of long-term outcome data demonstrating benefits beyond the prevention of deficiency [1].

Framing elevated protein intake as a population-wide recommendation, rather than as a conditional or subgroup-specific approach, blurs the critical distinction between meeting nutritional adequacy and pursuing optimization, a distinction that underpins the interpretation of dietary reference standards [2]. This lack of differentiation risks pathologizing normal dietary patterns and encouraging consumption levels that exceed requirements for health maintenance in the majority of individuals [1,3]. From a public health standpoint, it effectively repositions performance or therapeutic level targets as normative advice in the absence of robust long-term outcome data. Furthermore, within food systems that emphasize animal-based protein sources, such guidance may unintentionally promote greater intake of animal-derived foods [1,17]. This shift has potential consequences for cardiovascular risk, particularly if saturated fat intake increases, as well as for food affordability and environmental sustainability dimensions that are increasingly integral to comprehensive dietary guidance [19,20]. Table 1 illustrates the conceptual distinction between dietary strategies aimed at nutritional adequacy, optimization, and therapeutic application with respect to protein and carbohydrate intake.

Taken together, the current evidence supports higher protein intake in defined subgroups and under specific physiological conditions but does not justify its promotion as a universal target for the general population. Clearer differentiation between minimum requirements, conditionally optimal intakes, and therapeutic applications would improve the scientific precision and policy relevance of protein-related recommendations in the dietary guidelines. Overall, the evidence supporting higher protein intakes is strongest in specific physiological or clinical contexts, rather than as a universal recommendation for the general healthy population.

### 3.3. The Omission of Red and Processed Meat Limits

A notable omission in the 2025–2030 guidelines is the absence of a clear recommendation to limit or exercise caution regarding the consumption of red and processed meats. This is surprising given that the International Agency for Research on Cancer (IARC) has classified processed meat as a Group 1 carcinogen and red meat as a Group 2A probable carcinogen, with strong evidence linking their consumption to an increased risk of colorectal cancer [21]. At the same time, the guidelines encourage higher overall protein intake and present red meat as a prominent protein source in the accompanying visual guide. This creates an important policy inconsistency. On the one hand, the guidelines promote increased protein consumption, yet on the other they highlight a food source that has been associated with carcinogenic risk without providing any clear advice regarding recommended frequency, portion size, or potential health implications. This omission is particularly concerning because it does not differentiate between meeting protein requirements and the possible long-term health consequences of relying on certain high-risk protein sources. By failing to acknowledge this well-established body of evidence, the guidelines overlook a significant public health consideration and weaken the preventive health objectives that national dietary recommendations are intended to support.

### 3.4. Full-Fat Dairy and the Food Matrix Hypothesis

An expanding body of evidence has begun to challenge the long-held view that dairy fat uniformly increases cardiometabolic risk, contributing to the emergence of the “food matrix hypothesis.” This framework suggests that the health effects of foods cannot be adequately understood by examining isolated nutrients in isolation, but instead reflect interactions among nutrients, food structure, and processing characteristics [1,22]. Within this context, several meta-analyses of prospective cohort studies have shown that fermented dairy products particularly yogurt and certain cheeses are associated with neutral or modestly protective associations with cardiovascular disease, type 2 diabetes, and all-cause mortality, effects that appear largely independent of fat content [23,24]. These observations have been interpreted as evidence that fermentation-related bioactive peptides, probiotic activity, calcium–fat interactions, and the physical structure of dairy foods may mitigate the adverse lipid effects traditionally attributed to saturated fat [22,25].

Nevertheless, while the food matrix hypothesis represents a meaningful conceptual advance, the supporting evidence is heterogeneous and highly dependent on the specific dairy product. Metabolic effects vary substantially across dairy foods, and the neutral or potentially protective associations observed for fermented products cannot be extended to dairy fat as a whole [24]. In contrast, butter and cream foods that lack the microbial activity and structural features of fermented dairy have consistently been associated with less favorable lipid responses, particularly increases in LDL cholesterol when consumed alone or used to replace unsaturated fat sources in the diet [26,27]. These lipid changes are clinically meaningful, given the well-established causal role of LDL cholesterol in the development of atherosclerotic cardiovascular disease [20,28].

Consequently, the broad endorsement of full-fat dairy within the dietary guidelines, without clear differentiation between fermented and non-fermented products, risks oversimplification and conceptual overreach. Although emerging evidence supports moving beyond a simplistic low-fat versus full-fat framework, it does not warrant extending findings from yogurt and cheese to all full-fat dairy foods [22]. Framing full-fat dairy as generally acceptable may also unintentionally encourage higher consumption of butter, cream, and other non-fermented dairy fats, potentially weakening parallel recommendations to limit saturated fat intake to less than 10% of total energy [20]. Overall, the more favorable cardiometabolic evidence for full-fat dairy appears to apply mainly to fermented products and should not be interpreted as a general endorsement of all full-fat dairy foods.

From a policy and implementation standpoint, greater precision is needed. Clear differentiation between fermented and non-fermented dairy products, guidance on portion size and substitution context, and explicit placement of full-fat dairy within dietary patterns rich in unsaturated fats would improve alignment with established principles of cardiovascular risk reduction [1,27]. In the absence of such qualification, the current framing risks conflating context-specific evidence with population-level endorsement, increasing the likelihood of misinterpretation in clinical practice, public health communication, and food policy.

The guideline recommendation to limit saturated fat intake to less than 10% of total energy is consistent with long-standing strategies for cardiovascular disease prevention and reflects broad scientific agreement regarding lipid-mediated atherogenic risk [20]. Evidence from controlled feeding studies, metabolic ward experiments, and dietary substitution analyses consistently shows that replacing saturated fats with polyunsaturated fats leads to meaningful reductions in LDL cholesterol and, importantly, in cardiovascular event risk [27,28]. These findings are biologically plausible given the established causal role of LDL cholesterol in atherosclerotic cardiovascular disease and have been replicated across a range of populations and dietary settings. As a result, the strategy of replacing saturated fats rather than eliminating them entirely represents one of the most robust and consistently supported causal relationships in nutrition science [20,27].

Presenting butter and beef tallow as acceptable “healthy fat” options alongside olive oil introduces a clear conceptual inconsistency within the guidelines. Olive oil particularly extra-virgin olive oil is rich in monounsaturated fatty acids and bioactive polyphenols and has been consistently linked to favorable lipid profiles and reduced cardiovascular risk in both observational studies and randomized dietary interventions [29,30]. By contrast, butter and beef tallow are predominantly sources of saturated fat and lack comparable evidence supporting cardioprotective effects. Although debate continues regarding whether the metabolic effects of saturated fat vary by food source, the overall direction of substitution effects, namely that replacing saturated fats with unsaturated fats improves cardiovascular risk markers and clinical outcomes remains well established and largely uncontested [20,28].

The lack of explicit guidance on frequency of consumption, portion size, and substitution context further amplifies this inconsistency. In the absence of clear qualification, framing saturated animal fats as broadly acceptable may inadvertently encourage the replacement of unsaturated fats with saturated fats rather than the intended reverse, thereby weakening public health messaging around fat quality and cardiovascular risk reduction [20,27]. This framing creates a significant risk of public misinterpretation, fostering the misconception that “natural” fats like those in butter and tallow are inherently healthy and can be consumed without restriction, thereby ignoring the well-established negative impact of excessive saturated fat on blood lipid profiles [18,24]. This concern is particularly relevant also in media and consumer-facing interpretations of dietary guidance, where nuanced distinctions between “occasional inclusion” and “healthy choice” are frequently lost [1].

Such framing may also erode decades of cardiovascular disease prevention efforts by obscuring the central importance of fat quality and displacement effects within overall dietary patterns, despite strong causal evidence linking saturated fat-induced increases in LDL cholesterol to atherosclerotic cardiovascular disease [20,28]. Greater specificity is therefore essential for policy clarity and effective implementation. Explicitly positioning butter and beef tallow as optional, infrequent components of dietary patterns otherwise rich in unsaturated fats rather than as interchangeable alternatives to olive oil would enhance internal coherence and better align recommendations with established causal evidence [27,28]. Without such clarification, current messaging risks undermining one of the most evidence-based recommendations in nutritional epidemiology and may contribute to ongoing public confusion regarding the role of dietary fats in cardiovascular health [1]. Finally, the endorsement of energy-dense foods like butter and full-fat dairy, without explicit guidance on portion control and substitution context, also carries implications for total energy intake. Replacing less energy-dense foods with these options could inadvertently increase overall caloric consumption, working against public health goals for weight management.

### 3.5. Carbohydrate Restriction and Chronic Disease

Recognizing that lower-carbohydrate dietary patterns may be beneficial for individuals with chronic disease reflects a pragmatic appreciation of dietary heterogeneity and the limits of one-size-fits-all nutritional guidance. In people with type 2 diabetes, systematic reviews and meta-analyses of randomized controlled trials indicate that low and very low-carbohydrate diets can lead to short-term improvements in glycemic control, including reductions in HbA1c and higher rates of diabetes remission at around six months when compared with higher-carbohydrate dietary approaches [31,32,33]. These early improvements appear to be driven primarily by reductions in postprandial glucose excursions, lower insulin requirements, and short-term negative energy balance, rather than by carbohydrate restriction itself [34].

Nevertheless, the durability and broader applicability of these effects appear limited. At 12 months or beyond, differences in glycemic outcomes between low-carbohydrate diets and comparator approaches are largely diminished, with most meta-analyses showing convergence in HbA1c and fasting glucose across dietary patterns [29,31,32]. Reduced adherence over time, gradual relaxation of carbohydrate restriction, and compensatory increases in energy intake are likely key contributors to this attenuation, underscoring the challenges of sustaining such dietary approaches in free-living populations [35,36]. Distinguishing short-term metabolic responses from long-term dietary adherence is therefore essential when translating therapeutic dietary strategies into population-level recommendations.

Importantly, the cardiometabolic effects of low-carbohydrate diets are highly dependent on the quality of macronutrient substitutions. Evidence from meta-analyses and controlled trials shows that low-carbohydrate diets high in saturated fat are often associated with unfavorable changes in lipid profiles, particularly increases in LDL cholesterol, even when improvements in glycemic control are observed [32,37,38]. These lipid responses are clinically meaningful given the well-established causal link between LDL cholesterol and atherosclerotic cardiovascular disease [28]. By contrast, low-carbohydrate dietary patterns that prioritize unsaturated fats, plant-based protein sources, and minimally processed foods tend to attenuate adverse lipid effects and may result in more favorable overall cardiometabolic profiles [39,40].

These considerations have important implications for how dietary guidelines are interpreted. While it is clinically appropriate to acknowledge a potential role for lower-carbohydrate diets in the management of chronic disease, presenting these patterns without clear emphasis on fat quality, food sources, and long-term feasibility risks oversimplification. In addition, much of the supporting evidence comes from closely supervised interventions or highly motivated clinical populations, which limits straightforward extrapolation to the general population in the absence of sustained medical or dietary support [2,36]. A more evidence aligned approach would emphasize the reduction in total added sugar intake and discourage frequent consumption of sugar sweetened beverages and energy dense, nutrient poor sweets, without over specifying per meal limits that lack robust empirical backing [41]. Failing to clearly distinguish therapeutic dietary approaches from population-level recommendations may therefore increase the likelihood of misinterpretation and unintended shifts in habitual dietary patterns. However, this focus on carbohydrate restriction diverts attention from a more universally applicable principle that is “carbohydrate quality”. A robust body of evidence supports the replacement of refined carbohydrates and added sugars with high-fiber, minimally processed whole grains and legumes. This quality-focused approach, a cornerstone of dietary patterns like the Mediterranean diet, improves glycemic control, reduces insulin resistance, and lowers cardiometabolic risk without requiring the extreme and often unsustainable restriction of total carbohydrate intake [14,37]. By emphasizing restriction over substitution, the guidelines miss a critical opportunity to promote a more achievable and evidence-based strategy for the general population.

Taken together, the current evidence supports the selective use of lower-carbohydrate dietary patterns as a therapeutic option for certain individuals with chronic disease particularly those with type 2 diabetes when implemented under appropriate clinical guidance. However, the benefits of these approaches are neither consistent nor sustained across populations, and their cardiometabolic safety is highly dependent on dietary composition, substitution effects, and long-term adherence. More explicit qualification of these conditions within dietary guidelines would enhance scientific precision and improve alignment with established principles of cardiovascular risk reduction. The relative strength of evidence supporting the major guideline components is summarized in Table 2.

## 4. Limitations and Omissions

This review has several limitations. First, it was conducted as a critical narrative appraisal rather than a formal systematic review and therefore did not employ predefined inclusion criteria, formal risk-of-bias scoring tools, or quantitative synthesis methods. Second, much of the analysis relies on secondary syntheses, including systematic reviews and consensus reports, which may inherit limitations from underlying primary studies. Finally, as with any appraisal of complex policy documents, interpretive judgment is inherent in evaluating proportionality, coherence, and evidentiary scope. These considerations should be taken into account when interpreting the conclusions presented. Nevertheless, the review prioritizes high-level evidence sources and evaluates proportionality between evidence strength and policy framing, rather than re-estimating effect sizes.

Despite strong and consistent evidence linking higher dietary fiber intake to lower risks of cardiovascular disease, type 2 diabetes, colorectal cancer, and all-cause mortality, fiber receives relatively limited emphasis in the 2025–2030 Dietary Guidelines [16]. Large-scale meta-analyses have demonstrated clear dose–response relationships between fiber intake and cardiometabolic outcomes, with benefits observed across diverse populations and dietary patterns [16].

Hydration represents another area in which the guidelines rely on simplified behavioral messaging despite a relatively limited population-level evidence base. While recommendations to prioritize water intake and reduce consumption of sugar-sweetened beverages are well supported, hydration status itself remains poorly defined. There is no universally accepted standard for optimal hydration, nor a validated biomarker suitable for assessing hydration in free-living populations [42]. As with guidance related to protein intake and dietary fat, clearer distinction between evidence-based behavioral recommendations and context-dependent physiological targets would strengthen scientific precision and reduce the risk of misinterpretation in public- and media-facing communications.

A further cross-cutting limitation concerns the lack of operational clarity for several central concepts introduced in the guidelines. Terms such as “ultra-processed foods,” “healthy fats,” and “real foods” are used as organizing principles yet remain insufficiently operationalized for consistent application across policy, clinical counseling, research, and food labeling [5,43]. While classification systems such as NOVA are increasingly used in research, variability in definitions and thresholds complicates translation into regulatory or clinical frameworks [44]. Similarly, the term “healthy fats” encompasses heterogeneous food sources with markedly different fatty acid profiles and health effects, increasing the risk of selective interpretation and inconsistent implementation. Table 3 provides a summary of the inconsistencies within the guidelines.

Given that habitual fiber intake remains well below recommended levels in many countries, the relatively limited emphasis placed on fiber represents a missed opportunity to reinforce one of the most consistently supported, food-based public health messages in nutrition science [45]. The lack of clearer quantitative targets or stronger integration of fiber-rich foods within guideline messaging guidelines stands in contrast to the robustness of the underlying evidence base.

A further critical omission is the insufficient integration of environmental sustainability into the core dietary recommendations. While a shift toward minimally processed, plant-forward eating patterns aligns with both human health and reduced environmental impact, the guidelines do not explicitly frame their advice within this context. The endorsement of higher population-wide protein intakes and a more permissive stance on full-fat dairy and animal-based fats, without clear qualification regarding their source, carries potential environmental consequences. Animal-sourced foods, particularly red and processed meats, are associated with substantially higher greenhouse gas emissions, land use, and water consumption compared to plant-based protein sources [46,47]. By omitting this dimension, the guidelines miss an opportunity to cohere with global calls for sustainable food systems, such as those outlined in the EAT-Lancet Commission report. Integrating sustainability considerations would not only align dietary guidance with planetary health but also reinforce the recommendation to limit red and processed meat intake-a message that is currently underemphasized. Failure to address this nexus risks presenting a partial view of a “healthy diet” that is disconnected from the long-term viability of the food systems upon which public health depends.

Beyond nutrient-specific considerations, the guidelines also implicitly assume widespread access to minimally processed foods and high-quality dietary options. This assumption overlooks the substantial influence of socioeconomic factors, including food cost, neighborhood food environments, and broader structural inequities, on dietary behavior [1,17]. A large body of evidence indicates that dietary patterns rich in minimally processed foods, lean proteins, fruits, vegetables, and whole grains are often more expensive and less accessible for socioeconomically disadvantaged populations even if they can promote planetary and environmental health. In the absence of complementary structural or policy-level support, such guidance may inadvertently widen existing health disparities [19]. Without explicit recognition of these constraints, dietary recommendations risk remaining aspirational rather than realistically actionable for substantial segments of the population.

Taken together, these omissions point to a broader tension within the guidelines between simplification and scientific precision. While simplified messaging may improve public engagement, insufficient specificity risks obscuring evidence hierarchies, weakening implementation, and potentially exacerbating health inequities. Addressing these cross-cutting limitations through stronger emphasis on dietary fiber, explicit consideration of socioeconomic constraints, and clearer operational definitions would enhance both the scientific coherence and real-world applicability of the dietary guidance.

Physical activity represents an additional area in which the 2025–2030 Dietary Guidelines recognize a strong evidence base but fall short of meaningful integration with dietary recommendations. Although regular physical activity is appropriately emphasized as a cornerstone of health, dietary guidance, particularly regarding higher protein intake and carbohydrate restriction, is presented largely in isolation from physical activity status. This is despite substantial evidence that exercise modifies protein requirements, insulin sensitivity, and overall cardiometabolic risk profiles [48].

## 5. Conclusions

The 2025–2030 Dietary Guidelines represent a welcome shift away from narrowly nutrient-centered advice toward a clearer focus on whole foods and the reduction in ultra-processed products, an area where scientific evidence is both strong and consistent for promoting both human and environmental health. However, this level of evidentiary rigor is not applied uniformly across all recommendations. Population-wide targets for higher protein intake and a more permissive framing of saturated animal fats extend beyond what the current evidence can reliably justify for the general population, given that most robust benefits of higher protein intakes are observed in older adults, individuals undergoing weight loss, and specific clinical populations. Likewise, the lack of clear distinction between foundational nutritional requirements and strategies intended for specific subgroups or therapeutic contexts increases the potential for confusion, selective interpretation, and inconsistent implementation in both clinical and public health settings. Addressing all issues discussed in the limitations section will require guideline processes to more clearly differentiate between population-wide and context-specific recommendations, to apply transparent evidence hierarchies consistently across topics, and to provide operationally meaningful definitions and quantitative targets where robust evidence exists. Such refinements have the potential to preserve scientific rigor, reduce misinterpretation, especially in media and commercial environments, and enhance the real-world public health impact and equity of future dietary guidelines.

## Figures and Tables

**Table 1:** Nutritional adequacy, optimization, and therapeutic dietary strategies.

Category	Primary Goal	Evidence Base	Target Population	Example
Nutritional adequacy	Prevent deficiency	Balance studies, cohorts	General population	Protein RDA (0.8 g/kg/day)
Optimization	Enhance function	Short- to medium-term RCTs	Older adults, athletes	Higher protein for lean mass
Therapeutic intervention	Disease management	Clinical RCTs	Type 2 diabetes, obesity	Low-carbohydrate diets

**Table 2:** Strength of evidence supporting major components of the 2025–2030 Dietary Guidelines.

Guideline Component	Primary Evidence Base	Evidence Consistency	Appropriate Scope	Critical Comment
Reduction in ultra-processed foods	Cohorts, RCTs, umbrella reviews	High	Population-wide	Strong convergence of epidemiological and experimental evidence
Added sugar reduction	RCTs, cohorts	High	Population-wide	Per-meal limits lack methodological justification
Protein ≥ 1.2 g/kg/day	RCTs in older adults, weight loss trials	Moderate	Conditional	Evidence extrapolated beyond clinical contexts
Full-fat dairy	Prospective cohorts, food matrix studies	Moderate	Context-dependent	Benefits mainly for fermented products
Saturated fat < 10% energy	RCTs, substitution analyses	High	Population-wide	Strong causal LDL-mediated evidence
Low-carbohydrate diets	Short-term RCTs	Moderate	Therapeutic	Benefits attenuate over time

**Table 3:** Internal consistencies and tensions within the 2025–2030 Dietary Guidelines.

Guideline Domain	Recommendation	Evidence-Based Consideration	Potential Issue
Saturated fat	<10% energy	Strong LDL-C causal evidence	Undermined by permissive framing of animal fats
Dairy foods	Full-fat dairy acceptable	Benefits mainly for fermented products	Risk of overgeneralization
Protein intake	1.2–1.6 g/kg/day	Evidence strongest in subgroups	Conflates adequacy with optimization
Carbohydrate intake	Lower-carbohydrate patterns acceptable	Short-term benefits only	Therapeutic evidence extrapolated
Dietary fiber	Limited emphasis	Strong dose–response evidence	Missed public health opportunity

## Data Availability

No new data were created or analyzed in this study. Data Sharing is not applicable to this article.
